# Beliefs about mental health in incarcerated males: a qualitative interview study

**DOI:** 10.3389/fpsyt.2023.1242756

**Published:** 2023-09-14

**Authors:** Line Elisabeth Solbakken, Svein Bergvik, Rolf Wynn

**Affiliations:** ^1^Department of Clinical Medicine, Faculty of Health Sciences, UiT The Arctic University of Norway, Tromsø, Norway; ^2^Division of Mental Health and Substance Use, University Hospital of North Norway, Tromsø, Norway; ^3^Department of Psychology, Faculty of Health Sciences, UiT The Arctic University of Norway, Tromsø, Norway; ^4^Department of Education, ICT and Learning, Østfold University College, Halden, Norway

**Keywords:** lay beliefs, mental health, incarcerated, prison, attributions, ADHD, mental illness

## Abstract

**Introduction:**

Beliefs about mental health are shaped by the sociocultural context. Prisons have unique environmental and social features, and the prevalence of mental health problems in incarcerated populations is exceptionally high. These features make prisons especially interesting settings for exploring health beliefs. The aim of this study was to explore the conceptualizations of mental health and coping preferences in a prison environment.

**Methods:**

Individual in-depth interviews were conducted with fifteen incarcerated males from three prisons in Northern Norway. The design draws on central elements from Grounded Theory.

**Results:**

Mental health was perceived as distinct from mental illness by many of the participants. They coped with the prison environment by focusing on the things that gave them a sense of meaning and autonomy – this also formed their conceptualization of mental health. Furthermore, social interaction and activities were perceived as important to enhance and maintain mental well-being, however there were institutional barriers to using these coping strategies. The prison environment was integrated in the participants conceptualizations of mental health problems, and psychosocial stressors were emphasized in causal attributions. Biological and dispositional factors were less frequently mentioned. The participants preferred non-medical management for mental health problems and most displayed a reserved attitude towards psychotropic medications. The exception was attention-deficit hyperactivity disorder, for which they held neurobiological causal beliefs, together with a corresponding preference for medication as treatment.

**Conclusion:**

The main finding was a firm integration of the prison context in in the participants’ beliefs about mental health. We theorize that fusion of prison conditions and mental health beliefs were brought on by the processes of prisonization, observing mental distress in peers and attempts to protect self-esteem by externalizing the causes for mental health problems. Access to activities, social time, and “someone to talk to” were perceived to be crucial for improving and preserving mental health.

## Introduction

### Mental health among people in prison

Many of those who end up in prison have endured extensive childhood adversity and challenging living conditions (e.g., physical and mental abuse, parental neglect, low school attainment, economic disadvantage, and social exclusion) (1–4). The association between disadvantaged life circumstances and poor mental health is undisputed (5), and this relationship is also evident in the elevated rates of mental disorders in prison populations across the globe (1–3, 6–9). It is estimated that 92% of those imprisoned in Norway meet the criteria for one or more mental disorders (8). Studies find that incarcerated persons in Norway are reluctant to seek help for mental health issues and have low patient satisfaction with services provided by prison healthcare (10, 11). A recent report also suggests that the correctional system and mental health services fell short to adequately address the complex mental health and social needs of those imprisoned in Norway (12). The Norwegian government’s ten-year plan for improving mental health acknowledges that prison conditions, including the lack of human contact due to extensive isolation, are a central challenge in the Norwegian correctional system. Furthermore, the plan states that ensuring healthy prison environments and high-quality health services are essential for improving the mental health of those imprisoned (13).

### Formal definitions of mental health and illness

There are various ways of conceptualizing mental health (14, 15). A widely endorsed definition is that of The World Health Organization (WHO), that conceptualizes mental health as (14, p. 8):

A state of mental well-being that enables people to cope with the stresses of life, to realize their abilities, to learn well and work well, and to contribute to their communities. Mental health is an integral component of health and well-being and is more than the absence of mental disorder.

Thus, the WHO defines mental health as a positive concept distinctly different from mental disorders. This view corresponds with the positive psychology movement, where interventions typically aim to improve mental health by maximizing positive emotions, engagement, relationships, meaning and accomplishment, shortened with the acronym PERMA (16).

The WHO defines a mental disorder as disturbances of cognition, emotion regulation or behavior, which cause distress and impairment in critical areas of function such as social relationships and work (17). The broader term ‘Mental health problems’ incorporates temporary mental distress or milder emotional disturbances that may not meet the criteria for specific mental disorders (18). Essential in the conceptualization of mental disorders are causal attributions. According to the widely endorsed biopsychosocial model, mental health is determined by: “*(…) a complex interplay of individual, family, community and structural determinants*”(14, p. 19). Thus, individuals’ mental health is viewed as product of social and environmental influences that interacts with genetic and psychological processes (18). For instance, genes may predispose for mental disorders through their influence on personality, impacting how individuals respond to environmental stressors (19). The relative roles of genes and psychosocial causes vary between mental disorders, as some conditions are more heritable than others.

### Lay conceptualizations of mental health

“Lay theories” of mental health are implicit belief systems held by non-professionals about the causes, manifestations, consequences and cures for mental health problems (20). Lay persons beliefs about mental health are influenced by formal knowledge, as well as the sociocultural context and past experiences. Thus, the conceptualizations of mental health vary across different settings and populations (21–23). Lay peoples’ causal attributions of mental health problems involve a multitude of biological, psychological, and social explanations (22). The most frequent finding is that lay people tend to emphasize psychosocial stressors in their causal explanations of mental health problems (20, 22, 24). In later years there has been an increase in biological explanations for mental disorders among the public (25, 26). This is explained by the significant advances in neurobiological research, information campaigns and commercials for pharmacological treatments, which predominantly attribute mental disorders to chemical imbalances in the brain (27). Although biological explanations seem to be gaining ground, psychosocial explanations for mental health problems remain as the more commonly endorsed beliefs among the public (26).

### Consequences of beliefs about mental health

Causal explanations affect how people respond to mental health problems when encountering them (27–30). The relationships between causal explanations and preferences for coping with mental health problems are complex; however, there are some consistent findings. Since biological explanations imply that the causes of mental health problems are outside personal control, they deflect self-blame for such challenges (20, 31). Perceiving mental health problems as caused by temporary and uncontrollable environmental stressors rather than reasons that are intrapersonal and stable can also protect self-esteem (32). Thus, attributions can sometimes be adaptive when adverse life events, such as mental health problems, are realistically explained by external causes or reasons outside of personal control.

Causal attributions are also relevant for coping with mental health problems. Leaning towards biological explanations is associated with intentions for seeking professional help (21, 33, 34) and with more positive attitudes towards psychotropic medications (35). Primarily attributing mental health problems to external stressors is associated with informal help-seeking (33, 34). Furthermore, attributing mental health problems to internal causes such as personal weakness is associated with decreased perceived need for professional help (36, 37). Studies have also found a robust general preference for psychotherapy over psychotropic medications in community samples (24, 35, 38), which corresponds with the predominance of psychosocial explanations among lay people.

### Beliefs about mental health among incarcerated individuals

Individual beliefs about health, coping and treatment are shaped by context and personal life experiences (20, 39). As such, the mental health beliefs among those incarcerated naturally reflect the culture of the society in which they were socialized and belong (40). However, beliefs may also be influenced by the significant life transition of imprisonment. “Prisonization” is a term for psychological changes as a response to the immense restrictions and demands of prison life. The process of prisonization involves changes in thinking, feeling and behavior, as the norms of prison life are incorporated in the habits of the individual (41). Among the hardships of prison life are experiencing loss of autonomy, separation from friends and family, lack of meaningful activities, a social climate marked by distrust, fear of victimization and strict institutional regimes (42). Since the rates of mental illness, suicides and self-harm are disproportionately high among incarcerated individuals (43), it follows that exposure to peers in mental distress must be considerable for people in prison. Several studies have found that incarcerated individuals believe that the prison culture and environment have a negative impact on their mental health (44–47).

Various factors in the prison context may shape conceptualizations of mental health of those imprisoned. A handful of studies have provided some insight into the beliefs about health and illness of incarcerated persons; however, the current knowledge base is scarce (46). Understanding how incarcerated persons conceptualize mental health and how they perceive different options for coping is helpful in the endeavor to meet their health needs. The aim of this study was to explore the following questions: How do incarceration and the prison context influence conceptualizations of mental health and illness? How do the beliefs of incarcerated persons influence their preferences for coping with mental health problems?

## Method

### Study context

Norway has a prison population of nearly 3,000 persons and one of the lowest incarceration rates of the world with 56 per 100,000 (48). The prison conditions are humane compared to most other countries (49). As the intended punishment is the deprivation of liberty, the living conditions in prison should otherwise approximate the conditions of society in general (50). However, the humane conditions of the Norwegian correctional system may not be reflected in the quality of life of incarcerated individuals in Norway, which has been found to be comparable to that of other countries (51).

### Ethics

The study design adheres to the Helsinki declaration of medical research involving human subjects (52) and to the Norwegian Correctional System’s Guidelines for Research. All participants gave written informed consent. The study was approved by the Data Protection Officer of the University Hospital of North Norway. The Norwegian Correctional System Region North, which is the legal authority responsible for the welfare of the participants, also approved the study. Approval was sought from The Regional Health Research Ethics Committee of Northern Norway, which deemed the project outside their mandate.

### Study design

This study takes on a relativist ontological position which holds that reality is subjective and acknowledges the existence of different individual interpretations of the same phenomenon. We also adhere to the notion that researchers and participants co-construct knowledge in the research process (53). The design draws on central elements from Grounded Theory (GT), such as initial coding and categorization of data, constant comparative inductive and abductive analysis, writing memos, theoretical sensitivity, developing a core category and theoretical integration (54). Theoretical sampling is uncommon in prison research (55), and ethical and practical consideration is a probable reason. The data itself was the starting point of the analysis, and conceptual categories were developed through iterative comparative analysis to develop an explanatory theory. Data collection ceased when no new insights were gained, and the categories were sufficiently explained.

### Participants and study settings

Fifteen incarcerated males from three prisons in Northern Norway took part in the study. Among the participants, two individuals did not hold a Norwegian citizenship, while thirteen were Norwegian citizens. The age of the participants spanned from the early twenties to the late sixties (M: 43.6 years; SD: 13.57). Thirteen of the participants were held in high-security wards, and two were held in low-security wards. Since there were few participants, it is necessary to withhold further details about their age, ethnicity, and sentences to preserve their privacy.

### Recruitment

The prison leaders in each prison appointed a contact person to assist the researcher throughout the interview process. The contact persons were a social worker, a reintegration coordinator, and a prison officer. The contact person responsible for the study placed posters within the prison wards, inviting individuals to participate. The posters contained brief and easy-to-read information about the study’s general aim, that the interviews would be audio-recorded, confidential, held at the prison healthcare ward, and last about 1 hour. The posters encouraged those who would like to take part in the study to request more information from the contact person. Individuals who expressed interest in participating received comprehensive written information about the study from the contact person. This information emphasized voluntary participation and the right to withdraw consent at any stage, whether before, during, or after the interviews. Notably, the participants were also ensured that information from the interviews would not be shared with correctional staff. All individuals who received the additional information actively volunteered to participate by notifying the contact persons. One of those who agreed to participate withdrew from the study because of health issues on the interview day. The contact person scheduled the appointments between the participants and the first author, who conducted the interviews. Before the interviews, the first author had no knowledge of the participants or their backgrounds. Each participant received verbal information about the study directly from the first author and subsequently provided written consent. Following the interviews, participants were encouraged to share their feedback, ask questions, and were reminded of their right to withdraw their consent if desired. The reimbursement of incarcerated people for participation in research is a debated topic. Given the relative deprivation of the prison environment, it has been argued that even small incentives may have the potential for an undue influence for participation in research. For this reason, we chose to abstain from providing incentives for participation in this study.

### Interviews

Individual in-depth interviews with the participants were conducted in Norwegian. The interviewer thoroughly described the aim of the study and the participants’ rights before the interviews began. The interviews took place in health wards and visitation rooms and lasted 60–90 min. Only the interviewer and the participants were present during the interviews, and the interviewer had a personal alarm connected to the guard room as a safety precaution. The interviews loosely followed an interview guide with open-ended questions about; mental health and illness, the prison culture, and help-seeking. This flexible approach allowed follow-up questions on the participants’ experiences and perspectives.

### Analysis

The audio-recorded interviews were transcribed verbatim in Norwegian by the first author. The analysis started with the authors reading the interviews and asking questions regarding the data. The coding was initiated after completing the first eight interviews. The constant comparison method was used to compare instances and explore patterns and contrasting perspectives. From the ninth interview, the coding and data collection were parallel processes, with the coding of the new interviews included in the analysis as soon as they were transcribed. The coding was performed by the first author applying the NVivo 12 software. In the analysis, transcripts were read line-by-line while systematically asking questions concerning the data. Coding in the initial phase encompassed labeling of segments of meaning ranging from a couple of sentences to small paragraphs. The most frequent and significant codes were used to organize data into conceptual categories in the next focused coding phase. The authors had several meetings to discuss the transcripts, tentative categories, and interpretations throughout the analytic process. Memos of ideas, analytical choices, and interpretations were used as a basis for this collaborative reflexive process. In the last analyzing stage, the authors interpreted and negotiated the results, and the final conceptual categories with representative quotes, were developed. After completing the analysis, the quotes included in this report were translated from Norwegian to English.

## Results

The overarching category from the analysis was the integration of prison living conditions in the participants’ beliefs about mental health. This core category was represented in the other categories and explained how incarcerated persons understood mental health and how the prison context formed their beliefs. The first main category, “Perceptions on mental health,” explained how the participants conceptualized mental health and mental health problems. The second main category was “Beliefs about the impact of imprisonment on mental health.” The third and last main category was “Beliefs about the management of mental health problems,” which described how the participants’ causal beliefs were connected to their preferences for treatment (Table 1).

**Table 1:** Emergent categories.

Core category: prison life is integrated in beliefs about mental health	
Main categories	Sub-categories
C1: perspectives on mental health	C1SC1: conceptualizing mental health C1SC2: conceptualizing mental health problems C1SC3: causal explanations of mental health problems
C2: beliefs about the impact of imprisonment on mental health	C2SC1: prison specific causes for mental health problems C2SC2: the association between prisonization and mental health
C3: beliefs about management of mental health problems	C3SC1: preferences for management C3SC2: attention-deficit hyperactivity disorder is the exception

### Characteristics of the participants

Many of the participants gave considerable autobiographical accounts by their own choice. The participants’ experiences varied. About a third of them served their first sentence in prison, while two-thirds had served one or more sentences prior to their current one. About a third had been institutionalized or imprisoned for a major part of their adult life and had no or very limited work experience. One participant self-identified as a ‘professional criminal’. Several of them had rather chaotic social backgrounds and described unusual and adversive childhood experiences and circumstances. Three participants said they doubted their own ability to live independently upon release. About a third had formal education beyond secondary school and had been living a family life with steady income and housing. All participants had experienced mental distress while in prison, and about half claimed to have one or more diagnosed mental disorders.

### C1: perspectives on mental health

#### C1SC1: conceptualizing mental health

The participants focused on various aspects in their conceptualization of mental health. A minority of the participants perceived mental health to be the same as mental illness and did not seem to be aware of the positive aspects of mental health. A few also perceived their mental health as a neutral internal framework influencing how they perceived the world. If their mental health was good, they would have positive emotions and an optimistic outlook, whilst mental health problems were associated with negative perceptions of situations and surroundings. Some also emphasized the mind–body connection and approached mental health from a more spiritual perspective. Ultimately, the most common conceptualization of mental health involved a sense of balance and well-being:

*It’s the health of the psyche. If you feel good about yourself, on the inside. If you function normally*. P13.

Some of the participants talked about how they could enhance their mental health and mentioned things like physical activity, social contact, work, school, creative activities, and other coping strategies:

*I must admit that it is tough. And if it had not been for my guitar, you know. I can play, and like, get some “therapy” from it. Letting things go through writing lyrics.* P3.

The participants explained that various activities gave them a sense of direction and distracted them from negative thinking. Spending time with peers and in activities led by prison officers was seen as positive by the participants, and many longed for more opportunities for social connection and growth. Interestingly, many also described how their view on mental well-being had changed during their prison time. Some spoke of how they had realized the value of coping strategies such as going fishing or visiting friends, which they could no longer do. Others said they had come to appreciate the little things in life within the prison walls. Examples were valuing prison officers who treated them with trust and respect or the joy of choosing and ordering their own groceries once a week. Some of the participants had even turned this new perspective into a coping strategy:

*You reach for the little things, and you hold on to them. I believe that with my experience, behind bars, for such a long time. I value trivial things more than people in the outside community do.* P1.

Another participant said that complaining about things created a sense of community among the people at the ward:

*We complain about trifles. That it takes too long to get feedback from prison health on the application forms. That the coffee at the ward is in low supply, it is trivial, really. But we can get worked up about it, and we do. Over trifles. Grumblings and humor. It’s what we have in here, it’s what we live by.* P15.

While acknowledging that the prison setting severely limited personal choices and that services were inadequate due to limited resources, some of the participants emphasized that focusing on the things that were obtainable and within personal control could enhance adjustment and the sense of meaning:

*You have opportunities. Even if everything looks dark. And you cannot see the light in the tunnel. But, if you really, really want it. And you decide that you are going to make it, you can. Try to make use of the chances and opportunities you have, it is not much, but still, it’s doable*. P7.

In summary, the participants’ view on mental health was holistic and multifaceted. For some, adapting to prison life led to a revelation about the importance of social relationships and meaningful activities. They found that mental well-being could be enhanced by appreciating the little things, seeking companionship, reframing one’s circumstances and working on an accepting attitude.

#### C1SC2: conceptualizing mental health problems

In their definitions of mental health problems, most participants mentioned changes in thoughts, feelings and behaviors. The most noted and obvious sign of mental health problems in others was self-isolation, characterized by not participating in meals, choosing not to go to the prison yard and staying in the cell rather than seeking company with others in the common areas:

*First and foremost, I’m thinking that people have challenges, that make their everyday lives difficult. Uhm. Trouble with themselves […]. You become passive; you isolate yourself. And there are lots of thoughts that are destructive or suppressive*. P9.

Although the participants were asked to define mental health problems in general, most chose to speak about their own experiences of mental distress and illness before and during prison. Several participants mentioned they had symptoms of ADHD and various anxiety-related problems such as panic attacks, flashbacks from traumatic experiences and extensive worrying and rumination. Two of them also described their own experiences with substance use disorders, personality disorders and bipolar disorder. Most common among the participants were descriptions of depressive symptoms in themselves or others; however, they rarely used the label depression. Instead, they described the symptoms:

*It is dark. Everything is hard. Uhm. Nothing brings joy. You do not see the small positive things in your everyday life. Your focus is more on the negative stuff.* P15.

Common for all the participants was how they experienced their mental health problems as inseparable from their social environment. Many also talked about the influence of the prison setting on the mental health of their peers and how one must be robust to endure the hardships of prison life:

*You must be mentally strong to handle this. Not everyone does. It’s, It’s*… *Uhm. I’ve seen many that, like, “go apeshit” and smash everything in their cell to pieces, or that have even tried taking… I know about people who have taken their own lives here, in this prison. Not everyone can pull through.* P7.

Many also described a worsening of their mental health caused by various stressors in the prison environment. Exemplified in this quote by Participant 4 who according to himself, had good mental health prior to imprisonment:

*I believe it is caused by loss of control. I have a lot of nightmares. Traumatic experiences from earlier in life re-appear. It’s tormenting me! And I have NOT been struggling with this until now.* P4.

Years earlier, he had been at the scene of an accident with multiple fatalities but had coped well with his experiences before imprisonment. He believed the stress associated with losing control and adapting to the prison environment triggered flashbacks and nightmares.

#### C1SC3: causal explanations of mental health problems

The participants perceived mental health problems as caused by many factors, with a clear focus on the social determinants of health. They mentioned experiences of parental neglect, social exclusion, poverty, violence, sexual abuse, discrimination, and war. Some participants also pointed out that many who end up in prison have been exposed to a disproportionate amount of environmental risk factors for developing mental illness. Participant 13 said that his time in prison had altered his perspectives on mental health and criminality:

*I have come to understand that people have totally different starting points, and that things are not black and white (…) There are many that have had a hard time growing up. They had parents who sexually abused them, or that had a substance use disorder, or maybe both. And quite a few have been institutionalized almost their entire life, along with having a diagnosis or two, ADHD, or something else.* P13.

Intrapersonal factors such as dispositions or maladaptive thinking patterns were rarely mentioned by the participants. Furthermore, biological explanations of mental illness did not stand strong among the participants. Only a fourth of the participants mentioned the role of genetics, saying that mental illness tended to run in families:

*It may have been experiences from early life; it can be trauma. Some of it can be hereditary. […] The parents can have mental disorders, and the children get the same illness. I’ve seen that happen.* P9.

### C2: beliefs about the impact of imprisonment on mental health

#### C2SC1: prison-specific causes for mental health problems

Although the participants were aware of social determinants, they mainly attributed mental health problems in themselves and fellow incarcerated to the initial shock of imprisonment and the continuous hardships of prison life. Hence, prison living conditions were integrated into the participants’ definitions of mental health problems. Observing signs of mental distress and illness in others was experienced as ever-present in the prison community by many of the participants. Although they were not explicitly asked about their experiences with peers who had mental health problems, many of them spontaneously shared detailed stories of suicides, self-harm, trashing of inventory, arson and violent acts committed by incarcerated persons whom they believed to be in severe mental distress. Some participants said that while the prison officers were followed up after incidents, those imprisoned either received minimal follow-up or were left to deal with their experiences by themselves.

*The hopelessness you observe. In the end I think you do not give a shit, really. You’re not going to ask [for help]. So many people are self-harming and, and in the most severe cases, taking their own lives. And I understand it. That’s the worst part. There are only empty promises. You get a glimmer of hope, and then it slowly disappears again…* P4.

Almost all the participants mentioned the vast amount of time spent isolated in their cells as a substantial cause for mental distress, and some also explained that lack of meaningful activities and isolation gave room for unhealthy ruminations. Many argued that their autonomy was severely limited and perceived this as damaging. Moreover, some stated that the forced passivity harmed their sense of self-worth and outlook in life. About half of the participants described physical health problems which they claimed were not attended to by the correctional service and prison health and named this a cause of mental distress. They also explained barriers for contact with friends and family on the outside, such as phone calls being monitored, as a burden. In sum, all the participants claimed that prison conditions were damaging to the mental health of themselves and their peers (Table 2).

**Table 2:** C2SC1: prison-specific causes for mental health problems.

*In the weekends you are locked in by half past seven. And locked out by half past eight the next morning. It’s awful!* P12 *You have a lot of time to think about stuff. Negative stuff. It’s easy to get caught in a negative spiral here.* P15 *You are just a number. You feel powerless. You are degraded to the level of a young boy.* P3 *You lose yourself bit by bit every day, and you gradually turn into a zombie. And the bitterness you feel is ever-growing.* P13 *My physical health influences mental health to a large degree. It makes my situation worse.* P6 *When I talk to my parents or my daughter or something, it’s very constrained. You do not want to talk about personal stuff, and that is mentally harmful.* P9

#### C2SC2: the association between prisonization and mental health

The participants widely endorsed the perceived connection between prisonization and mental health. Many associated the process of prisonization with deteriorating mental health. When the interviewer asked a participant if he talked about mental health with his fellow incarcerated, he responded:

*Yes, we do. We talk about the way we become by being here. We lose our memory; we forget which day it is. We talk about how we can see that others are prisonized, and we are teasing them: “Now you are damaged like the rest of us.” And we observe the tragedies as the years go by. Those who have been here the longest are really damaged.* P13.

Some participants experienced anxiety symptoms in social settings, which they attributed to the prisonization process. These symptoms were especially prominent when they were on furlough from prison. They described how they believed everyone was watching them and how they felt trapped and panicked in situations where they could not get away. They also spoke of physical symptoms like sweating, trembling and a pounding heart. The participants seemed surprised by the perceived strength and physical nature of the symptoms they experienced:

*Something happens with our brain. When you are isolated in a restricted community for long periods. And then you are let out in the real world (…). Everyone is looking at you. “He is acting weird or stressed-out. And why is he all sweaty’n’stuff” But it’s not, it’s not real. They are not looking at you. It’s in your head.* P7.

Anxiety symptoms related to prisonization seemed to be more acceptable to talk about among the incarcerated than other mental health related problems. Some of the participants appeared to believe that prisonization caused permanent damage to the mental health and cognition of themselves and their peers, making rehabilitation and a meaningful and lawful life after prison less achievable:

*There are incredibly many who are having a tough time mentally. And the mental things we are struggling with here. Sadly, I believe it will follow you for the rest of your life. It does not disappear as you walk out through the gates. In my opinion, people that serve a continuous sentence longer than a year in prison get permanently damaged.* P8.

### C3: beliefs about management of mental health problems

#### C3SC1: preferences for management

The participants shared a clear preference for psychosocial strategies when they talked about coping with and seeking treatment for mental health problems. Despite the barrier of trust issues, seeking support from other incarcerated was seen as the most available means of taking care of one’s mental health in prison. Peers were an essential source of support because they had first-hand knowledge about issues specifically related to the experience of imprisonment:

*Finding someone you trust; someone you can talk to. It does not necessarily have to be about mental illness, but to share your thoughts (…) You can have issues that are related to having a tough time, like challenges in relationships with those on the outside, or relations to people in here, how one should behave towards the officers or other inmates, if one has problems with anyone (…) and then they can give you input on how they would act and what they think about the situation.* P11.

Some participants expressed disappointment with being offered medications instead of access to other means of coping with imprisonment and associated mental distress. They wanted more social time, services, and activities that they perceived to be beneficial for coping and mental health. Although some participants acknowledged that medications effectively numbed negative emotions related to the initial shock of imprisonment, several participants also said they would prefer managing distress by talking to healthcare personnel rather than taking medications. However, “someone to talk to” was perceived as challenging to obtain, whereas access to medications was seen as unproblematic by most of the participants:

*During the first week they asked me if I wanted sleeping pills, or other medicines, to get me through the initial shock. And I said that I had appreciated having someone to talk to the first few days, and that I wanted to continue with that. I did not want to become a worse person than I was when I came in.* P14.

Medications were perceived to reduce symptoms of mental distress; however, they were not considered to be a cure or an effective long-term solution for mental health problems by several of the participants:

*Medications for anxiety and such, it’s a disservice really. It is damn good in the moment, but it only creates more problems. Sure, you are numb for a little while, but then your problems pile up.* P11.

Several participants expressed critical attitudes towards the perceived excessive use of psychotropic medications in prison, and some had also seen undesirable effects of such medications in their fellow incarcerated. A few of the participants also believed that psychotropics had harmful effects:

*If you can avoid medications, then avoid them! Uhm, I know what those medications do to your brain, and I have declined. Cutting receptors, and they never heal. At least if you are using it for longer periods, it creates permanent damage. And that’s why I’m not keen on psychotropic drugs. I do not use them.* P9.

#### C3SC2: attention-deficit hyperactivity disorder is the exception

The participants’ attitudes towards Attention-deficit hyperactivity disorder (ADHD) differed from other mental health conditions in several ways. ADHD was seen as more acceptable in the prison culture; it was a diagnosis which was pursued by several of the participants. One of the participants said he had a formal diagnosis of ADHD, while four of the other participants claimed to have undiagnosed ADHD and said they were arguing for a diagnostic assessment or re-assessment:

*I’ve been in and out of prison since my teens. I’ve spent years in prison. And it’s been all right. I can guarantee that I have ADHD. But I have not been assessed for it (…) There’s always a reason for ending up here. And there are often underlying problems, whether it is substance use or ADHD, or acting out. Having a low threshold for resorting to violence in a pub or ending up in a fight. Something like that, which is the underlying problem.* P8.

For ADHD, medications were clearly preferred over psychosocial treatments. A few of the participants said they had been self-medicating with amphetamine before prison, and that they wanted prescriptions for ADHD-medications to address their symptoms:

*There are so many that get an ADHD-diagnosis that are faking it, and like, that’s not my thing. I know that I’m struggling with something – restlessness in my body, inside me. I make it better by doing amphetamine.* P3.

Participant 9, who did not have ADHD himself, explained that he believed ADHD is an acceptable reason to seek help from a psychologist in prison:

*The majority of those who receive treatment from a psychologist say it’s because of ADHD. And like, it’s not always true, it is often used as an excuse. It’s an acceptable diagnosis (…) It is also a justification for ending up in prison: “I’m not to blame for this situation. I have ADHD. I’m not able to control myself”* P9.

Participant 9 goes on to explain that you must go through a formal assessment from a psychologist to get an ADHD-diagnosis which is necessary to get medications:

*The preoccupation with medications is extreme in here. So is the trading of medications (…) (…) it is immensely popular to get hold of central stimulants. You see and hear things, without really taking part in the conversation.*

When the interviewer asked why central stimulants were so popular, the participant responded:

*Those who do not need it. They get the same reaction as from amphetamines. That’s why it’s so popular. And we who do not use such things, we do not want others to do it, because it can affect the peace and atmosphere in the wards, and such.*

The idea of ADHD as an explanation for criminal activity and ending up in prison was also supported in the narratives of some of the other participants who did not claim to have ADHD.

## Discussion

This study delves into the beliefs about mental health and illness among fifteen incarcerated persons across three correctional facilities in Northern Norway. Employing an exploratory qualitative approach, the study captured nuanced insights into the participants’ perspectives on mental health and potential remedies for mental health challenges. The core finding was that the experience of imprisonment was integrated into the beliefs about mental health of the participants. The participants described how their beliefs about mental health had changed through the processes of adapting to the prison culture (i.e., prisonization) and observing the mental distress of peers. Attributing mental health problems to stressors in the prison environment rather than to internal causes could be an effective strategy for protecting self-esteem. This attributional tendency may also have influenced the participants’ beliefs about mental health. The results engender suggestions for improving mental healthcare within correctional settings.

### Conceptualization of mental health

Although the participants’ understandings of mental health varied, most perceived it as a concept describing a state of well-being or as a framework for how we perceive and act in the world. Some of the participants understood mental health to be the same as mental illness; however, most participants perceived mental health as a positive concept. The participants also shared how they found meaning in activities and relationships in their daily lives, which they related to well-being and coping. Several participants’ narratives indicated that their well-being and mental health concepts had changed since entering prison. Some described how they found gratification in trivial things, which they would not have appreciated to the same degree on the outside. Others described how they longed for things they could no longer do, which they had only realized were valuable for their mental health after imprisonment. Making the most out of their little autonomy and freedom, such as selecting and ordering groceries and making food of their liking with others at the ward, was perceived as essential for their well-being. Results from other studies have also found that the perception of having choices and positive reframing is associated with well-being and better quality of life among people in prison (56, 57). Thus, the participants’ intuitive and active definition of everyday routines and activities as autonomous and meaningful supported the idea that such strategies could be positive for coping with imprisonment and enhancing well-being.

### Conceptualization of mental health problems

Mental health problems were conceptualized as disturbances of thoughts, emotions, and behaviors. Most of the participants associated mental health problems with distress, and some also mentioned impaired functioning. These definitions align well with the general definition of mental disorders in the ICD-11 and other studies of lay perspectives on mental health problems (22, 58). Specific diagnostic labels were used by some participants, while others described symptoms such as low mood, irritability, stress, anger, helplessness, hopelessness, and self-isolation. Social determinants of health, such as social exclusion, low income, experiencing violence, sexual abuse, and war were emphasized by the participants in their causal explanations of mental health problems which corresponds neatly with the research literature (19). Nearly all the participants recounted stories from their own, or fellow incarcerated individuals’ lives and related these narratives to their causal attributions of mental health problems. Hence, the participants demonstrated awareness of the relative disadvantage of mental health of incarcerated individuals. For some participants, this insight had developed through their interactions with others in prison, and the influence of the sociocultural context was evident in the participants’ beliefs about mental health problems. While there were individual variations, the participants had a relatively good understanding of what constitutes mental health problems.

### Causal attributions of mental health problems in prison

Psychosocial explanations are predominant in lay theories about mental health problems, and the emphasis on social and environmental stressors by the participants of this study were broadly similar to the views of the general population (24, 26). What sets this study apart from studies of lay theories in other populations were the causal explanations for mental health problems which were distinctly tied to the prison context. A tendency for normalizing and underestimating symptoms of mental health problems has been reported in other qualitative studies on lay theories of mental health problems (22). In our study, the participants seemed to underline the perceived severity of their mental distress, and at the same time they forcefully blamed the system and the conditions they were living under. About half of the participants said prison conditions worsened their pre-existing mental health problems. This is consistent with other studies which suggest that while the onset of mental health problems might predate imprisonment, the frustration and disempowerment caused by prison conditions may contribute to the risk of deteriorating mental health (44, 59, 60).

While there were some examples of internal causal attributions for mental health problems such as having maladaptive thought patterns, most of these internal states and processes were perceived to be brought on by prison circumstances. The tendency for external explanations by the participants in our study corresponded with other studies that have reported that incarcerated individuals saw mental health problems as inseparable from living conditions in prison (45–47). The observed pattern was not exclusive to incarcerated individuals, as an inclination to endorse external causes such as painful life experiences has also been observed in hospitalized patients with various mental disorders (61). Although some participants mentioned genetics, other biological explanations for mental disorders such as chemical imbalances were not endorsed by the participants, with the exception of ADHD. This followed the tendency to underestimate biological factors, as reported in other studies (20, 61). The results were consistent with attributional theory, which proposes that people tend to externalize the responsibility for negative events to protect their self-esteem (62). In fact, attributing negative life events to internal and uncontrollable factors has been associated with depressive symptoms in incarcerated individuals (63). The emphasis of external explanations for mental health problems in combination with attending to positive and controllable aspects of their everyday lives, can be viewed as adaptive cognitive reframing of a stressful situation by the participants.

### Beliefs about prisonization and mental health

The process of prisonization – or adapting to the prison environment, were perceived by the participants as damaging to their mental health. A few of the participants believed that the psychological symptoms related to prisonization were caused by changes in their brains. Interestingly, these were examples of truly biopsychosocial lay perspectives since they assumed an interaction between social, psychological, and biological causes for mental health problems. While the assumed effects of prison environments on brain functioning may seem extreme, there is emerging evidence of decline in executive functions, particularly reduced emotion-regulation and self-control after 3–4 months in prison (64, 65). Furthermore, deficits in emotion-regulation are a known risk factor for developing various mental disorders (66). Hence, there is some empirical support for the participants’ subjective experiences of prisonization and their beliefs about the impact on mental health. Although the participants attributions were specific for the prison situation, they took on a stable attributional characteristic since the consequences of prisonization on mental health are believed to endure after release. Holding pessimistic beliefs about the consequences and duration of various mental health problems is associated with a poor outcome (67, 68). Accordingly, simply believing that the effects of imprisonment on mental health are persistent could have negative long-term effects on the well-being and motivation for rehabilitation in incarcerated individuals.

### Observing distress in others influenced beliefs about mental health

Knowing and interacting with others who are experiencing mental illness may influence beliefs and attitudes about such challenges (69). Many of the participants gave detailed descriptions of more severe expressions of mental distress and illness among their fellow incarcerated such as self-harm, suicides, violence, trashing of inventory, arson, and delusions. Based on the participants’ accounts, exposure to other peoples’ mental suffering in prison was rather extreme, considering the apparent prevalence and severity of the incidences. This finding corresponds with reports from other studies which have found that the exposure to self-harm and suicides is exceptionally high in prison (70). The participants described how experiencing these severe incidences negatively affected their own mental health. This aligns with a study that found relatively higher levels of mental health issues including, anxiety, depression, hopelessness, and more severe suicidal ideations, among those who have had contact with another’s fatal or injurious self-harming behavior in prison (71). In our study, experiencing the despair of others was believed to cause hopelessness and a sense of disempowerment, and particularly when some claimed that they were left to deal with their experiences on their own. Gates et al. argued that more knowledge is needed in order to reduce the harmful effects of experiencing the mental suffering of others in prison (43), and our findings support this notion. Experiencing the suffering of peers also seemed to exacerbate beliefs that prison conditions influenced mental health negatively.

### Preferences for coping with mental health problems

Lay beliefs about the causes of mental health problems influence help-seeking and treatment preferences (28, 29). In accordance with their emphasis on external stressors, the participants expressed a preference for non-medical interventions for mental distress; exercise, excursions, creative activities, social interactions, organized peer-groups and talking with healthcare professionals were seen as health promoting and effective management of mental distress and illness. Nearly all participants in this study emphasized the importance of support from their families and fellow incarcerated for mental health. The highlighting of social contact is in line with another study which found that incarcerated individuals who made use of active coping by sharing negative feelings with their social network had better mental and physical health than those who did not (57). While some of the participants shared their emotions with others, most emphasized the positive effects of shared activities and social company with their peers. In summary, their preferences for “social cures” are in line with findings from qualitative reports on lay beliefs of mental health problems in other populations (22).

### Beliefs about medications for mental health problems

The participants beliefs and attitudes towards psychotropic medications in our study were unexpected. Psychotropics were perceived as very common in prison, which is supported by prevalence studies of medications in correctional settings (72). A few of the participants expressed positive attitudes to psychotropic medicines, and some also mentioned that medications might be necessary for more severe mental disorders. However, most participants expressed negative attitudes towards using medications for mental health problems. Many related their attitudes to first-hand experiences, and some also said they had seen negative behavior, which they attributed to use of prescribed psychotropics in their fellow incarcerated. While some studies have found that access to psychotropic medications is perceived to be insufficient by incarcerated individuals (73), the access to psychotropics was perceived as unproblematic by most of the participants in our study. However, several participants said they preferred activities and someone to talk to over medications but that the access to such services was inadequate. Other studies have also found that non-pharmacological treatments are preferred by people in prison but perceived as relatively inaccessible compared to medications (74, 75). The reserved attitudes of many participants towards psychotropics form a contrast to reports of excessive demand for medication from incarcerated individuals (73). This finding could represent atypical attitudes among the participants of this study. An alternative explanation is that the perceived high demand for psychotropics in prison is influenced by other factors, such as their trading value and potential for misuse, rather than an actual belief in their efficacy for treating mental health problems. Considering that psychotropics are efficient for reducing symptoms of various mental health conditions, exploring beliefs and providing adjusted information may reduce objections and enhance treatment adherence.

### Perspectives on ADHD in a prison context

The prevalence of ADHD is high in prison populations (76, 77), and recent studies suggest that ADHD is underdiagnosed and undertreated in prison (78–80). The proposed treatment gap for ADHD may have societal consequences, since treatment with psychostimulants in incarcerated individuals is effective (81), and associated with a reduction in recidivism rates (81, 82). For incarcerated persons, receiving an ADHD diagnosis could serve as an explanation for why they ended up in prison. The participants notion about an association between ADHD and criminal behavior is supported in the research literature (80, 83). Their perspectives on ADHD included autobiographical narratives of symptoms from an early age, a perceived need for self-medicating, and beliefs of the effectiveness of psychostimulants for treating their condition. Taken together, this suggests that several of the participants believed that ADHD had neurobiological causes, which could explain the desirability of the diagnosis since it deflects blame for deviant behavior. The potential for misuse of ADHD-medications, and their trading value may also be a motivational factor for wanting an ADHD diagnosis, which was also indicated in our data. It has been noted that healthcare personnel perceive some incarcerated individuals as deceitful in their attempts to obtain medications for reasons other than medical needs (73). The risk of addiction, misuse and trading of psychostimulants necessitates a cautious prescription practice in a prison context (72, 80). Despite such challenges, a combination of psychostimulants, psychoeducation and psychological, educational and occupational programs have been recommended treatments for incarcerated individuals suffering from ADHD (79). While stigma and lack of help-seeking may explain treatment gaps for many mental health problems, our results suggest that such factors might not be as relevant for ADHD. This raises the question of whether the primary causes of undertreatment of ADHD in prison populations is found at a system level.

### Implications for mental healthcare in prison

Jordan argues that: *“situation specific and culturally responsive mental health care is a must; context is crucial”* (84, p. 33). The findings of this study align with prior research, indicating that individuals in prison perceive mental health problems as interconnected with the prison environment (45–47). Primarily attributing mental health problems to external prison-specific causes may alleviate self-blame. The downside is that it may leave individuals less empowered to take personal responsibility for improving their mental health, for instance, by choosing effective coping strategies or adhering to formal treatment. Cognitive behavioral therapies that aim to change dysfunctional thinking and behavior patterns are common, and predominantly rely on internal attributions for mental health problems. In the course of treatment, health professionals and users of healthcare services may hold different causal attributions, which can make mutual understanding and communication more demanding (85). Patients who perceive that their causal attributions of mental health problems are congruent with the focus of therapy may find the interventions more helpful and desire future treatment to a greater degree than those who perceive dissimilarities (86). In clinical practice, exploring and acknowledging patients’ beliefs and adjusting treatment to the challenging living conditions in prison may be necessary for enhancing engagement with treatment.

In recent years, the significance of improving the mental health status of those incarcerated has been increasingly recognized within the public health domain (87). Mental health is more than the absence of mental disorders. Experts agree that merely focusing on preventing mental disorders and treating their symptoms is an inadequate strategy for improving the mental health of imprisoned populations (88–90). Due to limited resources in mental health services, a one-sided focus on mental illness imposes the risk that only those with the most severe problems are attended to (89). Thus, interventions reach more people and are more effective if they also aim to enhance positive mental health (91). This implies that mental health promotion in prison is a commitment which extends beyond the scope of prison health services (89). Our findings suggest that interventions aimed at improving well-being align closely with incarcerated individuals’ beliefs and preferences. Providing incarcerated persons with opportunities of maintaining social relationships, and experiencing personal growth through work, education, and meaningful activities are crucial elements for achieving and promoting mental well-being in prison. Improving the services for prisoners in these areas also corresponds with the Norwegian governments’ mental health priorities for the correctional system (13). However, the participants underlined the significant structural barriers that must be addressed to achieve these objectives. Substantial progress is needed to ensure prison conditions that truly foster mental health.

### Limitations

It is important to grasp the limitations of this study when considering its implications. The participants in this study were self-selected, and may have had more knowledge, interest, and willingness to talk about mental health issues than the average person in prison. In a strict sense, the results of this study do only apply to the fifteen individuals from three prisons in Northern Norway who took part in the study. We do not propose that the results generalize to the prison population in Norway, or in other countries. That said, our findings correspond well with, extend, and elaborate on studies from other correctional settings. We believe that we have provided sufficient information about the study context, participants and methodology for researchers and mental health professionals to evaluate the relevance of the results in other settings.

## Conclusion

This study explained how life experiences and living conditions in prison were firmly and inseparably integrated into the perspectives on mental health in incarcerated persons. Factors in the prison environment were seen as the direct cause of mental health problems, while improved access to social time and meaningful activities were perceived to foster mental well-being.

## Data availability statement

The datasets presented in this article are not readily available due to privacy concerns, the data are not publicly available. Requests to access the datasets should be directed to rolf.wynn@gmail.com.

## Ethics statement

The requirement of ethical approval was waived by the Regional Health Research Ethics Committee of Northern Norway because they deemed the study outside their mandate. The studies were conducted in accordance with the local legislation and institutional requirements. The participants provided their written informed consent to participate in this study.

## Author contributions

LS designed the study, did the interviews, analyzed the data, drafted the manuscript, revised the manuscript, and approved the final manuscript. SB analyzed the data, drafted the manuscript, revised the manuscript, and approved the final manuscript. RW designed the study, analyzed the data, drafted the manuscript, revised the manuscript, and approved the final manuscript. All authors contributed to the article and approved the submitted version.

## Funding

The study was in part funded by The North Norway Regional Health Authority (Helse Nord RHF). The Publication Fund of UiT The Arctic University of Norway supported the payment of the APC.

## Conflict of interest

The authors declare that the research was conducted in the absence of any commercial or financial relationships that could be construed as a potential conflict of interest.

## Publisher’s note

All claims expressed in this article are solely those of the authors and do not necessarily represent those of their affiliated organizations, or those of the publisher, the editors and the reviewers. Any product that may be evaluated in this article, or claim that may be made by its manufacturer, is not guaranteed or endorsed by the publisher.
